# Dissecting *Phaseolus vulgaris* Innate Immune System against *Colletotrichum lindemuthianum* Infection

**DOI:** 10.1371/journal.pone.0043161

**Published:** 2012-08-17

**Authors:** Paula Rodrigues Oblessuc, Aline Borges, Bablu Chowdhury, Danielle Gregório Gomes Caldas, Siu Mui Tsai, Luis Eduardo Aranha Camargo, Maeli Melotto

**Affiliations:** 1 Department of Biology, University of Texas, Arlington, Texas, United States of America; 2 CENA, Universidade de São Paulo, Piracicaba, SP, Brazil; 3 Departamento de Fitopatologia, ESALQ, Universidade de São Paulo, Piracicaba, SP, Brazil; Ghent University, Belgium

## Abstract

**Background:**

The genus *Colletotrichum* is one of the most economically important plant pathogens, causing anthracnose on a wide range of crops including common beans (*Phaseolus vulgaris* L.). Crop yield can be dramatically decreased depending on the plant cultivar used and the environmental conditions. This study aimed to identify potential genetic components of the bean immune system to provide environmentally friendly control measures against this fungus.

**Methodology and Principal Findings:**

As the common bean is not amenable to reverse genetics to explore functionality and its genome is not fully curated, we used putative Arabidopsis orthologs of bean expressed sequence tag (EST) to perform bioinformatic analysis and experimental validation of gene expression to identify common bean genes regulated during the incompatible interaction with *C. lindemuthianum*. Similar to model pathosystems, Gene Ontology (GO) analysis indicated that hormone biosynthesis and signaling in common beans seem to be modulated by fungus infection. For instance, cytokinin and ethylene responses were up-regulated and jasmonic acid, gibberellin, and abscisic acid responses were down-regulated, indicating that these hormones may play a central role in this pathosystem. Importantly, we have identified putative bean gene orthologs of Arabidopsis genes involved in the plant immune system. Based on experimental validation of gene expression, we propose that hypersensitive reaction as part of effector-triggered immunity may operate, at least in part, by down-regulating genes, such as *FLS2-like* and *MKK5-like*, putative orthologs of the Arabidopsis genes involved in pathogen perception and downstream signaling.

**Conclusions/Significance:**

We have identified specific bean genes and uncovered metabolic processes and pathways that may be involved in the immune response against pathogens. Our transcriptome database is a rich resource for mining novel defense-related genes, which enabled us to develop a model of the molecular components of the bean innate immune system regulated upon pathogen attack.

## Introduction

Common bean (*Phaseolus vulgaris* L.) is one of the most important staple foods in developing countries and it has been suggested as a model species for studying legume crops [1], [2]. Dry bean production can be drastically reduced by fungal pathogens. For instance, anthracnose caused by the fungus *Colletotrichum lindemuthianum* (Sacc. & Magnus) Briosi & Cavara, can result in total yield loss depending on the cultivar and environmental conditions [3], [4]. In fact, anthracnose has been rated within the top 10 most important fungus-caused disease in plants based on its scientific and economic relevance [5]. Development of genetically resistant plants can minimize the occurrence of this disease and maximize crop production. To this end, it is crucial to understand the mechanisms by which plants recognize the presence of a pathogen as well as the molecular changes that occur in the host cell upon pathogen infection. Studies in model pathosystems have revealed that virulence factors interact with host proteins triggering defense responses in an incompatible reaction [6]. The same virulence factors may act as suppressors of defense responses leading to disease development in a compatible reaction [7], [8].

**Table 1:** Differentially expressed ESTs identified in both libraries based on their enzyme codes.

Up-regulated genes						
Enzyme Code^a^	Enzyme name	Cellular localization	No. ESTs Control tissue	No. ESTs Inoculated tissue	p-value	RE^b^
EC:5.3.1.6	ribose-5-phosphate isomerase	chloroplast	0	3	0.09	1.06
EC:3.5.1.1	asparaginase	unknown	0	3	0.09	1.06
EC:2.3.1.0	unknown	membrane	0	3	0.09	1.06
EC:1.1.1.95	D-3-phosphoglycerate:NAD+ oxidoreductase	chloroplast, nucleus, cytosol, mitochondria	0	3	0.09	1.06
EC:3.2.1.39	endo-1,3- *β* -glucanase; callase	vacuole, endomembrane system	1	5	0.09	1.02
**Down-regulated genes**						
**Enzyme Code** a	**Enzyme name**	**Cellular localization**	**No. ESTs Control tissue**	**No. ESTs Inoculated tissue**	**p-value**	**RE** b
EC:4.1.1.39	ribulose-bisphosphate carboxylase	chloroplast	144	75	0.002	−2.66
EC:2.7.11.25	MAPKKK; MEKK	cytoplasm, plastid, plasma membrane	8	1	0.05	−1.30
EC:2.1.2.1	Serine and threonine aldolase	cytosolic ribosome, membrane, apoplast	5	0	0.07	−1.15

The enzyme codes were established using KEGG as part of the Blast2GO suite. Statistical significance was calculated with the Fisher’s exact test (p≤0.1).

a The ESTs names corresponding to enzyme code are described in Table S1.

b RE  =  relative expression values were obtained by -Log_10_ of p-values for the up-regulated transcripts and by Log_10_ of p-values for the down-regulated genes, according to Zhou *et al.* [79].

The function of the resistance and avirulence genes involved in the *P. vulgaris*–*C. lindemuthianum* interaction and the kinetics of plant defense have not been fully described yet, hindering research aimed to understanding this specific pathosystem. Genetic studies indirectly indicate that this pathosystem operates in a gene-for-gene manner [9]. Different anthracnose resistance genes/locus can be found clustered in the bean genome. For instance, resistance genes against *C. lindemuthianum* and other diverse pathogens such as *Uromyces appendiculatus* (causative agent of bean rust) and *Pseudomonas syringae pv. phaseolicola* (causative agent of halo blight), were identified at the end of the linkage group B4 [10]. The anthracnose resistance genes, named *Co*, are single dominant genes in the host and specific race/cultivar interaction exists [11]. Among these, *Co-4* located on a gene cluster at the linkage group B8 [12], confers the broadest-base resistance to anthracnose [13] making it very attractive for genomic studies with direct application to agricultural problems. For instance, the bean breeding line SEL 1308 carries the *Co-4^2^* gene for anthracnose resistance [12] and when inoculated with 34 selected races of C. lindemuthianum chosen to represent a diverse sample of the pathogen population, SEL 1308 demonstrated a resistance index (RI) of 97% [13]. An incompatible interaction between a bean cultivar carrying a *Co* gene and an avirulent race of *C. lindemuthianum* leads to the formation of necrotic spots in the host tissue typical of hypersensitive reaction (HR) and localized host-cell death [14]. Hypersensitive reaction is characterized by an oxidative burst that occurs by the generation of reactive oxygen species (ROS) resulting in programmed cell death [15]. This process is an early response in many pathosystems and in common bean it appears to be dependent on three components: an exocellular peroxidase, an extracellular alkalinization that occurs due to calcium influx and potassium efflux, and release of a substrate [16], [17].

**Figure 1:**
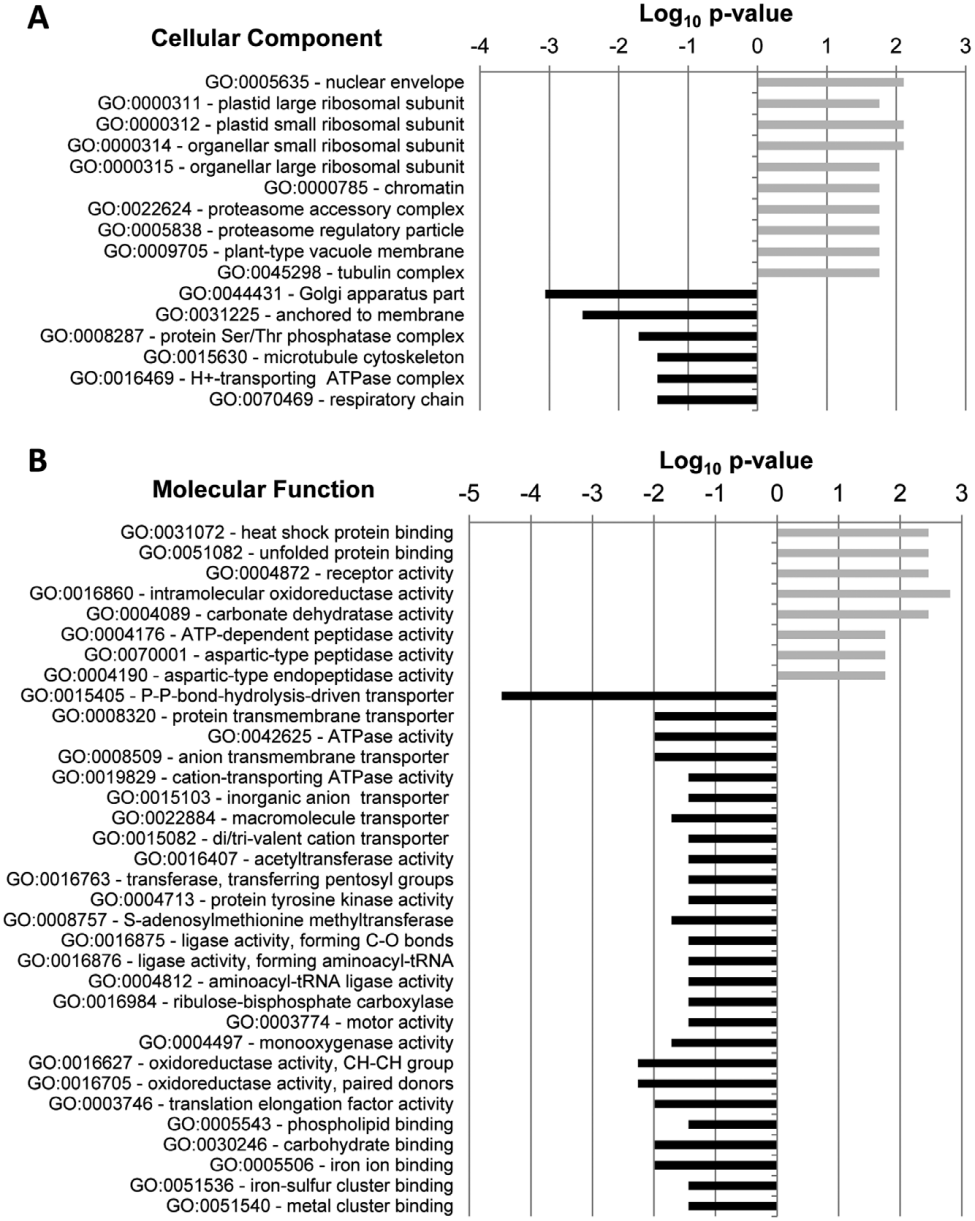
Gene Ontology categories uniquely identified in either EST collections using the AgriGO Singular Enrichment Analysis (SEA). Processes identified only in fungus-inoculated tissue are considered up-regulated by fungal infection (grey bars) and processes identified only in mock-inoculated tissue are considered down-regulated (black bars). Statistical significance was detected with the Fisher’s exact test (p-value ≤0.05) and data points are indicated as Log_10_ of the p-value. (A) GO terms in the cellular component category. (B) GO terms in the cellular function category. GO terms in the biological processes category are indicated in Fig. S1.

The *P. vulgaris*–*C. lindemuthianum* pathosystem has been genetically studied in attempts to clone resistance genes using map-based approach [18], [19], [20], to isolate resistance gene analogs [10], [21], [22], and to assess the expression of resistance gene candidates [12], [23], [24], [25]. The infection process and establishment of compatible interaction have also been well characterized at the cytological level [26], [27], [28]. Differential accumulation of specific defense-related transcripts such as mRNA for polygalacturonase-inhibiting protein (PGIP) and pathogen related (PR) proteins, during compatible or incompatible interaction between common bean and *C. lindemuthianum* has also been reported [29], [30]. More recently, genes encoding glutamine synthetase (GS1α), formate dehydrogenase, and EF-hand calcium-binding have also been implicated in immune response against *C. lindemuthianum* [31], [32], [33]. A few gene expression studies using northern blot were also performed, showing the up-regulation of genes during the incompatible interaction [34], [35].

**Figure 2:**
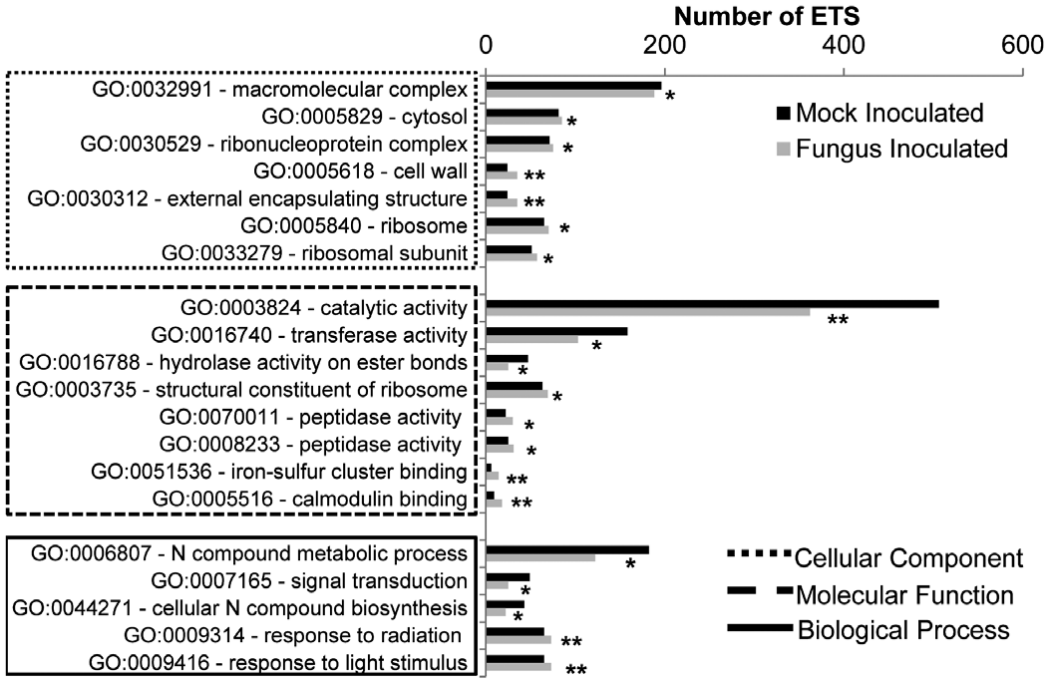
Gene Ontology categories identified in both EST collections using the AgriGO Singular Enrichment Analysis (SEA). Data points represent the number of ESTs in each collection that was placed in each GO category. Statistical significance of the relative abundance of ESTs in each collection was detected with the Fisher’s exact test (* = p≤0.1, **p≤0.05).

As genomic and genetic resources (*e.g*., extensive gene annotation and mutant lines) are scarce for common beans, transcriptome analyses may be a fast and cost-effective way to find differentially regulated genes under stress ultimately leading to the characterization of key steps in the defense response. Expressed sequence tag (EST) libraries are useful resources for mapping of expressed genes [36], [37] as well as for providing data for comparisons with related species such as soybean [38] or other plant model organisms such as Arabidopsis and rice [24]. Some EST libraries were developed to identify bean genes related to response to biotic and abiotic stresses such as bean rust [39], drought, low soil phosphorus, and high soil aluminum toxicities [40], [41], [42], as well as genes expressed during the development of pods, leaves [42], and seeds [43].

**Table 2:** Differentially expressed transcripts represented by ESTs identified in both libraries.

Up-regulated transcripts							
AGI number^a^		Annotation	Cellular localization	No. ESTs Controltissue	No. ESTs Inoculated tissue	p value	RE^d^
ATCG00470		ATP synthase epsilon chain (ATPE)	mitochondria	3	11	0.01	1.89
AT2G39730		Rubisco activase	nucleus, chloroplast	22	31	0.05	1.29
**Down-regulated transcripts**							
**AGI number**	**Annotation**		**Cellular localization**	**No. ESTs Control** **tissue**	**No. ESTs Control** **tissue**	**p value**	**RE^d^**
AT2G34430	Type I chlorophyll a/b-binding protein of Photosystem II		chloroplast thylakoid	83	14	<0.001	−9.50
AT5G54270	Type III chlorophyll a/b-protein complex of Photosystem II		chloroplast thylakoid	33	8	<0.001	−3.00
AT5G38430	Ribulose bisphosphate carboxylase		chloroplast thylakoid	55	20	<0.001	−2.70
AT2G05100	Type II chlorophyll a/b-protein complex of Photosystem II		chloroplast thylakoid	22	5	0.01	−2.22
AT3G61470	Type II chlorophyll a/b-protein complex of Photosystem I		chloroplast thylakoid	26	8	0.02	−1.82
AT1G61520	Type III chlorophyll a/b-binding protein of Photosystem I		chloroplast thylakoid	14	3	0.03	−1.55
AT1G67090	Ribulose bisphosphate carboxylase		chloroplast thylakoid	91	51	0.04	−1.40
AT1G15820	Light harvesting complex of photosystem II(LHCB6)		chloroplast: envelope, thylakoid, and thylakoid membrane; plastoglobule	20	7	0.08	−1.10
AT2G13360	Alanine glyoxylate aminotransferase (AGT1)		chloroplast stroma, peroxisome, plasma membrane	7	1	0.08	−1.08
AT1G08380	Photosystem I subunit O (PSAO)		chloroplast thylakoid	14	4	0.09	−1.03

The AGI number indicates putative Arabidopsis orthologs of bean transcripts identified with tBLASTX search against the TAIR10 database (E-value ≤1×10^−4^ was considered as significant). Statistical significance was calculated with the Fisher exact test (p≤0.1).

a The bean EST orthologs of each AGI number are listed in Table S2.

b RE  =  relative expression values obtained by −Log_10_ of p-values for the up-regulated transcripts and by Log_10_ of p-values for the down-regulated genes, according to procedures described by Zhou *et al.* [79].

In this study, we used EST libraries developed from seedling shoots inoculated or not with *C. lindemuthianum* [24] to examine overall changes in gene expression during the incompatible interaction between the resistant bean breeding line SEL 1308, which carries the *Co-4^2^* anthracnose resistance gene, and the avirulent race 73 [13]. We uncovered metabolic processes and pathways that may be involved in the common bean innate immune response against pathogens and develop a model representing key components associated in this interaction. In addition, we identified known and novel specific bean genes associated with immune response and experimentally validated gene expression inferred by bioinformatic analysis. Our result should provide insights for the developments of molecular tools (*e.g.* marker for differentially regulated genes) to be used in bean breeding programs as well as basic genetic information for functional annotation of the bean genome.

**Figure 3:**
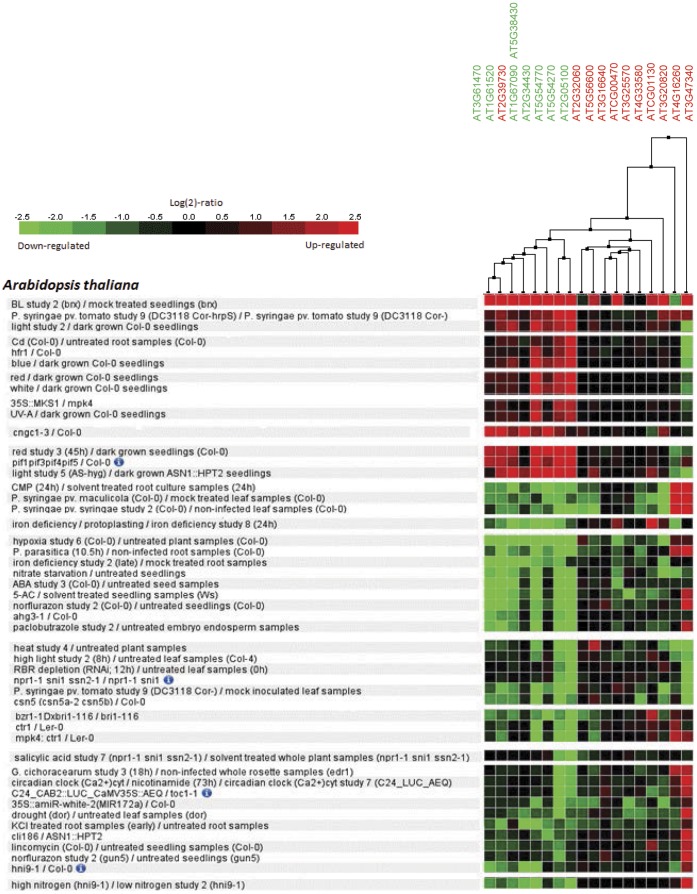
Patterns of expression of differentially expressed genes in fungus-inoculated tissue (p≤0.05). Expression of these genes in response to several other stresses was assessed through the publicly available microarray database, TAIR. Putative Arabidopsis orthologs of those bean genes were used to create a heat map obtained with the clustering tool of Genevestigator. Gene numbers in green and red colors indicate their down- or up-regulation in our study, respectively.

## Results and Discussion

### Gene Ontology Analysis of *Phaseolus vulgaris* Transcripts

To assess the overall changes in the transcriptional profile during the incompatible interaction between *P. vulgaris* and *C. lindemuthianum*, we compared two collections of ESTs; one collection obtained from two libraries (PVEPLE1 and PVEPSE2) constructed with control, mock-inoculated seedling shoots and another one obtained from a library (PVEPSE3) constructed with fungus-inoculated seedling shoots [24]. Fungal penetration in the host cell occurs within 54 hours post inoculation (hpi) and the proportion of affected cells increases over time [44]. Previous studies have shown that necrotic cells appear in bean leaves 72–96 hpi with an incompatible race of the fungus [44], when the cytoplasm of epidermal cells beneath appressoria appeared granular and pale brown in color [27] and the fungus complete its biotophic phase around 72 hpi [14], [44], [45]. Therefore, we collected plant tissue 65 hpi to construct the EST libraries, when the majority of host cells may be infected by the fungus and the HR is still ongoing.

**Figure 4:**
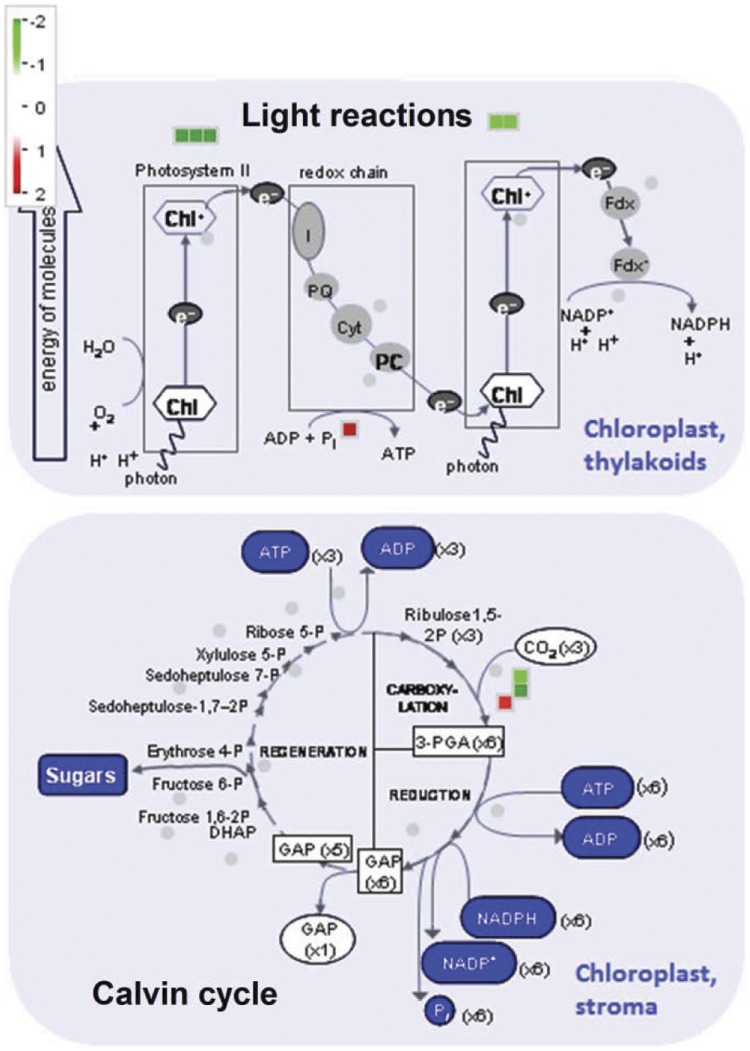
Cellular function overview of proteins encoded by photosynthesis-related genes differentially expressed upon fungus attack. Putative Arabidopsis orthologs of these genes (p≤0.05, Table 2) were used as input for Mapman analysis.

Initially, each EST was aligned (tBLASTX) against the non-redundant database using the Blast2GO suite [46] to assess the overall enzymatic activity affected by fungus infection (Table S1). Interestingly, 20% of ESTs from inoculated tissue and 13% of EST from mock-inoculated tissue had no hit to any sequence. We identified five enzyme categories that were more significantly abundant in the EST collection from fungus-inoculated tissue and three that were more significantly abundant (p≤0.5) in the mock-inoculated tissue (Table 1). Enzymes in two of these categories, 1,3 β-D-glucanase and MAPKKK, are well known to play important roles in plant defense against pathogens [47], [48]. Intriguingly, the gene encoding for a 1,3 β-D-glucanase was up-regulated while MAPKKK-encoding gene was down-regulated in fungus-inoculated tissue.

**Figure 5:**
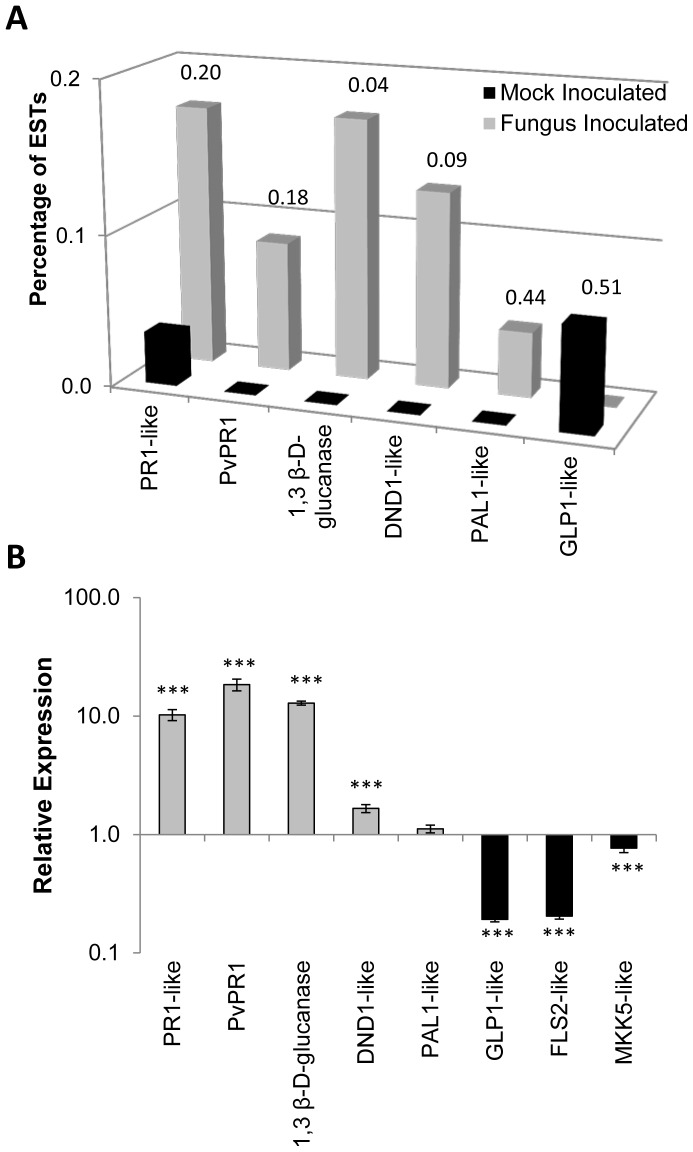
Expression level of selected gene used to validate the bioinformatic analysis of the bean EST collection. (A) Percentage of ESTs encoding the indicated protein in each EST collection obtained from mock- or fungus-inoculated tissue. Numbers on top of the bars indicate the p-value calculated with Fisher’s exact test. (B) Relative expression of the same genes assessed by RT-qPCR. Statistical significance was detected with the Student’s t-test in (*** = p<0.001).

As *Arabidopsis thaliana* (L.) Heynh has one of the most comprehensively annotated and manually curated genome, and in many cases supported by extensive experimental data, individual bean ESTs were aligned (tBLASTX) with Arabidopsis transcript sequences using the TAIR10 database (http://arabidopsis.org/) to enable functional comparison between our EST libraries. Out of 2,923 ESTs from mock-inoculated tissue and 2,232 ESTs from inoculated tissue, 2,489 (85%) and 1,843 (83%) showed significant similarities (E-value≤1×10^−4^) with Arabidopsis transcripts, respectively (Table S2). Putative Arabidopsis gene orthologs (AGI numbers) were used to identify GO numbers (Table S2) so that: 1) the overall changes in metabolic processes occurring in inoculated tissues could be identified through Singular Enrichment Analysis (SEA) using AgriGO [49]; 2) the relative expression of transcripts could be assessed in each EST collection to identify specific bean genes regulated upon inoculation; 3) regulation of specific genes by different stimuli, their location in the cell and their involvement in specific biochemical activities could be assessed; and 4) specific bean genes could be chosen to validate bioinformatics analysis using qPCR.

**Figure 6:**
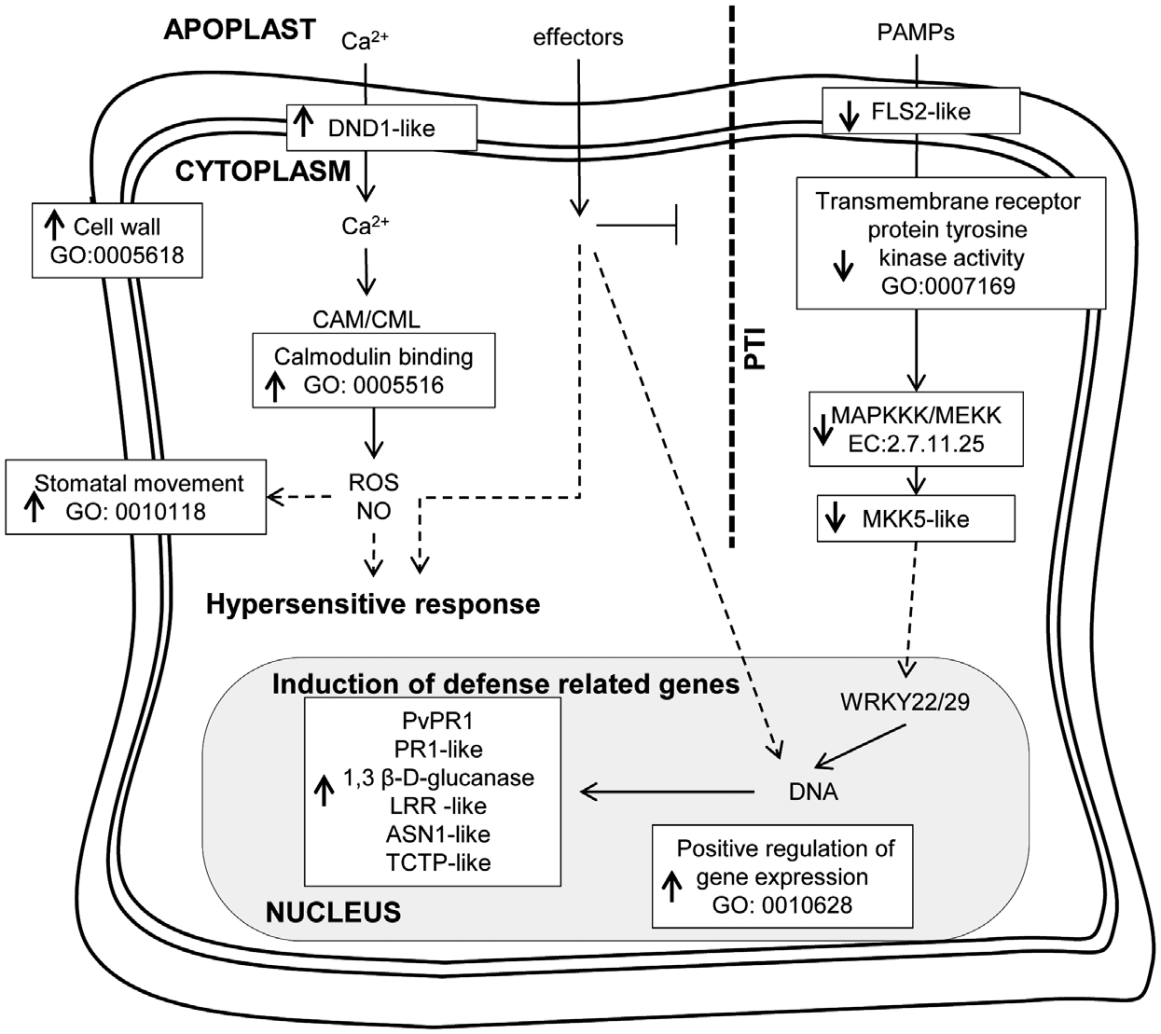
A model of the bean innate immune system. The proposed model represents key molecular components and metabolic processes known to be involved in plant-pathogen interactions based on KEGG, for which bean orthologs have been identified in our EST collection with significant statistical support (p≤0.05). Gene Ontology (GO) categories and Enzyme Codes (EC) inside boxes are differentially represented in the bean EST libraries (arrowheads pointing down represent down-regulation and arrowheads pointing up represent up-regulation). Continuous arrows represent established relationship between components of the pathway and intermittent arrows represent undirected relationship. Components of PAMP-triggered immunity (PTI) are depicted to the right of the diagram.

### Metabolic Processes in *Phaseolus vulgaris* Regulated upon *C. lindemuthianum* Infection

To identify metabolic processes that are specifically modulated by fungus infection, we annotated the bean transcripts using GO terms and identified both EST library-specific and differentially represented GOs. Overall, 842 GO terms were identified, out of which 134, 195, and 513 GO terms were within Cellular Component (GO:0005575), Molecular Function (GO:0003674), and Biological Process (GO:0008150), respectively (Table S3). Out of these, 136 GOs were found to be library-specific and significantly regulated (p≤0.05) indicating substantial metabolic changes in response to fungus (Fig. 1 and S1).

We have identified both well-known and novel metabolic processes involved in plant response and HR to fungal infection indicating that information from model plants such as Arabidopsis can be used to study less understood pathosystems. For instance, metabolic processes known to be involved in plant-pathogen interactions, such as the defense response to fungus (GO:0050832), regulation of defense response (GO:0031347), regulation of response to stress (GO:0080134), and stomatal movement (GO:0010118), were up-regulated in fungus inoculated tissue (Fig. S1). Likewise, up-regulation of response to cytokinin stimulus (GO:0009735) and ethylene mediated signaling pathway (GO:0009873) indicate that these hormones may also play an important role in common bean defense against *C. lindemuthianum*. Interestingly, jasmonic acid biosynthetic (GO:0031408) and metabolic (GO:0009694) processes, as well as response to gibberellin stimulus (GO:0009739) and abscisic acid mediated signaling pathway (GO:0009738) were down-regulated upon fungal infection (Fig. S1) highlighting the importance of hormonal control and cross-talk (antagonist or synergistic) in response to pathogen attack. Specific hormones may be required for resistance in some pathosystems and for susceptibility in others, which may be related to the pathogen life style (*i.e.* necrotroph or biotroph). Jasmonates (JA), for instance, are required for disease susceptibility of Arabidopsis and tomato plants to the biotroph bacterium *Pseudomonas syringae* [50], [51]. *Colletotrichum lindemuthianum* is a hemi-biotroph pathogen; thus it is not surprising that JA signaling is down-regulated in an incompatible reaction with the bean plant. Furthermore, components of the proteasome accessory complex (GO:0022624) and proteasome regulatory particle (GO:0005838) are over-represented in inoculated tissue. Plant hormone signaling is tightly regulated post-transcriptionally by the ubiquitin-proteasome system [52]; however, it is not clear how these two processes are connected in the bean-*C. lindemuthianum* pathosystem.

Oxygen and ROS metabolic process (GO:0006800) was also up-regulated suggesting that the bean plant modulates its metabolism for detoxification from ROS burst that occurs during HR [15]. Even though specific gene transcripts related to photosynthesis were down-regulated in fungus inoculated tissue, we observed an increase in gene products located in the plastid ribosome (GO:0000311, GO:0000312, GO:0000314, GO:0000315; Fig. 1A). The strong activity in the plastids could be explained by their involvement in ROS production.

Changes in transcription profile seem to be a key component occurring in plants under pathogen attack [39], [53] and it is known that this involves negative regulation of genes related to plant development in order to reallocate the resources to defense responses. This seems to be the case of common bean infected by *C. lindemuthianum*, as the abundance of transcripts encoding proteins associated with organelle fission (GO:0048285), cell cycle process (GO:0022402), pattern specification process (GO:0007389), post-embryonic morphogenesis (GO:0009886), and regulation of post-embryonic development (GO:0048580) were reduced in fungus-infected seedling shoots, while the abundance of transcripts encoding protein involved in positive regulation of biosynthetic process (GO:0009891) was significantly increased (Fig. S1). Furthermore, resources seems to be reallocated, at least in part, from up-regulation of catabolic process of lipids (GO:0016042), fatty acids (GO:0009062), glycine (GO:0006546), and serine family amino acids (GO:0009071).

We extended the GO enrichment analysis to identify quantitative differences between mock- and fungus-inoculated tissues (Fig. 2). Twenty GO categories were differentially represented in the two EST collections and supported by strong statistical significance. The high number of transcripts encoding calmodulin binding (GO:0005516) proteins observed in inoculated tissue agrees with the production of ROS during the cell immunity response as they are involved in Ca^+2^ signaling and redox homeostasis of the cell [54]. Furthermore, we observed an increase in transcripts encoding proteins located in the cell wall of inoculated tissue suggesting that activities at the cell wall may be an important component of bean defense against *C. lindemuthianum* as it has been reported in the *Populus* × *Melampsora* pathosystem [55]. These activities may include modification of cell wall material for resistance against penetration, pathogen recognition, and transport and secretion of defense compounds [56].

### Differential Expression of Specific Genes

To identify specific genes that were differentially expressed in fungus-infected tissue, we searched for library-specific ESTs. Each EST was aligned with Arabidopsis transcripts (TAIR10) using tBLASTX with a cut-off E-value of 1×10^−4^ (Table S2) and the number of ESTs in each collection that aligned to the same Arabidopsis gene transcript was determined (Table S4). Many ESTs aligned with the same Arabidopsis gene and were used to infer the level of expression of each bean transcript. The EST library constructed with inoculated tissue had 25 unique transcripts represented by three or more ESTs, whereas the library constructed with control, mock-inoculated tissue had 28 unique transcripts (Table S5). No function could be assigned to two gene transcripts found only in the EST collection from fungus-inoculated tissue and five gene transcripts observed only in the mock-inoculated tissue (Table S5). Interestingly, all but one was predicted to locate at the chloroplast. This fact points out that these seven genes potentially play a role in incompatible interactions, thus opening doors for the characterization of novel defense genes in common bean. Furthermore, transcripts identified only in fungus-inoculated tissue seem to be involved in response to abiotic stress, such as cold, starvation, and drought, as well as biotic stress, such as response to pathogenic fungus or bacteria. Genes involved in ubiquitination (E3 ligase), response to hydrogen peroxide and ion transport also appear to be up-regulated during incompatible interaction (Table S5).

We further identified genes with significant differential expression (p≤0.1) between the mock-inoculated and fungus-inoculated tissue (Table 2). Interestingly, transcripts encoding for ribulose bisphosphate carboxylase and chlorophyll a/b-binding proteins were down-regulated during fungal infection; however, the bean plant seems to compensate with up-regulation of rubisco activase (Table 2) and gene transcripts encoding proteins related to carbon utilization (GO:0015946, Fig. S1). Decrease in photosynthetic rates due to anthracnose infection has also been observed by gas-exchange analysis [57]. Similar changes have been demonstrated in different pathosystems. For instance, tomato plants inoculated with *P. syringae* pv. *tomato* also showed down-regulation of photosynthesis and chloroplast related genes within four hours of incubation, whereas transcription factors were up-regulated and protein turnover through the ubiquitation pathway and proteases increased [58].

To verify the possibility that differentially expressed genes between our EST collections are co-regulated by other types of stresses, we used their putative Arabidopsis orthologs to create a heat map of relative expression using public microarray data available at Genevestigator (Fig. 3). The 19 differentially expressed genes with a p-value ≤0.05 (Tables 2 and S5) have corresponding array probes. Consistent with our observation that ethylene mediated signaling may be up-regulated (Fig. S1), these genes are also regulated by ethylene in a similar manner as indicated by the microarray analysis of *ctr1-1* mutant plants [59]. Surprisingly, these genes are also regulated in the pathosystem Arabidopsis - *P. syringae* pv. *syringae* and *maculicola*, which represents a compatible interaction. This may be explained by the fact that most of these genes are involved in photosynthesis (Table 2) which is generally negatively affected by pathogens [57]. A complete list of treatments with similar and opposite gene expression patterns is depicted in Fig. 3.

As several transcripts differentially represented in our EST collections encode proteins possibly involved in photosynthetic pathways (Tables 2 and S5), we used their putative Arabidopsis orthologs to visualize the specific reactions these proteins catalyze and evaluate the overall metabolic shift in cells under attack by *C. lindemuthianum* (Fig. 4). The light-dependent reactions of photosynthesis seem to be down-regulated, suggesting that the cell could be depleted from ATP synthesized through the redox chain; however the cells seem to compensate by up-regulating the synthesis of ATP. Similarly, the abundance of the enzyme RUBISCO decreased in fungus-inoculated tissue, however the enzyme RUBISCO activase was up-regulated probably to restore the normal function of the carboxylation step of the light-independent reactions (Calvin cycle).

### Validation of Gene Expression using Quantitative PCR

Relative expression of specific bean genes determined by reverse transcriptase quantitative polymerase chain reaction (RT-qPCR) agreed with the gene expression patterns predicted by the bioinformatic analysis of the ESTs libraries (Fig. 5). Four up-regulated genes encoding for PR1-like, PvPR1, 1,3 β-D-glucanase, and DND1-like proteins (Fig. 5A), were annotated as being involved with defense response based on the function of their putative Arabidopsis orthologs (Table S6). Genes coding for PR1 proteins are usually used as markers for systemic acquired resistance (SAR), but their functional role has remained elusive [60]. *PvPR1* transcript has been originally identified in a cDNA library obtained from bean cell suspensions treated with *C. lindemuthianum* mycelium cell wall fractions [61] and is a putative ortholog of the MPL-like gene of Arabidopsis also implicated in plant defense. Endoglucanases, such as the 1,3 β-D-glucanase, can also contribute to defense response directly and indirectly, which can result in pathogen cell wall degradation and liberation of oligosaccharides that may act as endogenous signaling agents [47]. A transcript that encodes for a putative cyclic nucleotide regulated ion channel (DND1-like) was found only in the inoculated tissue. This type of proteins allows the passage of cations through the plasma membrane, such as the DND1 of Arabidopsis seems to be essential for triggering HR in a gene-for-gene dependent manner [62]. However, ATPase-coupled transmembrane ion transport activity, represented by GO:0016469 (Fig. 1A), GO:0042625 and GO:0019829 (Fig. 1B), seems to be down-regulated by fungal-inoculation.

The enzyme PAL (phenylalanine ammonium lyase) is known to be involved in plant defense against pathogens [63], [64], [65]. *PAL* mRNA was also induced in response to *C. lindemuthianum* during incompatible and compatible interactions, being stronger and faster during the incompatible interaction, with a maximum expression at 60 hpi [34]. However, we identified only one EST from fungus-inoculated tissue (65 hpi) that was annotated as encoding for a PAL1-like protein. Because this *PAL1-like* transcript sequence does not align perfectly with the one reported previously (accession number M11939) [66], it is possible that it belongs to a paralogous gene in the bean genome which is not involved in plant defense [65]. The low abundance of *PAL1-like* transcripts (gi|59938140) identified in our EST collection indicates that this particular gene is not regulated by fungus infection. This result was supported by RT-qPCR analysis (Fig. 5B) indicating that our EST collections are representative of the gene expression patterns identified by bioinformatics analysis.

GERMIN and GERMIN-like protein (GLP) have been implicated in defense against abiotic and biotic stress in plants [67]. Although GLP has an oxalate oxidase activity in monocotyledons, degrading oxalate to CO_2_ and H_2_O_2,_ which plays an important role in plant immunity, GLP does not appear to have oxalate oxidase activity in dicotyledons [68]. GLP was classified as pathogenesis related (PR) protein specifically induced during hot pepper defense response against viral infection [69]. *GERMIN-like* (*GLP1-like*) transcript was only found in the control, mock-inoculated bean tissue (two ESTs) suggesting down-regulation by fungus infection. Although the calculated p-value is 0.51 (Fig. 5A), RT-qPCR analysis confirmed the down-regulated of this gene (Fig. 5B). We therefore reasoned that the low statistical significance of EST abundance may be due to the fact that our EST collection does not represent the bean transcriptome to the saturation level and the identification of library-specific transcript (Table S5) represents a good source of gene candidates involved in defense against *C. lindemuthianum.*

### A Working Model for the Bean Innate Immune Response

The bioinformatic analysis of common bean ESTs and experimental validation of gene expression revealed the transcriptional changes and underlying metabolic processes that occur during an incompatible interaction between *P. vulgaris* and *C. lindemuthianum*. In addition to identifying genes that were specifically expressed or repressed under the stress imposed by this fungus we were able to identify overall cellular activities modulated by this fungus. As this pathosystem represents an incompatible reaction resulting in hypersensitive response, we searched for specific molecular components involved in plant innate immunity, both PTI (PAMP-Triggered Immunity) and ETI (Effector–Triggered Immunity) [70]. In fact, we found the common bean counterparts of several steps of the Arabidopsis immune system against the bacterial pathogen *P. syringae* pv. *tomato* (Fig. 6). As suggested for model pathosystems [70], it seems that ETI, also characterized by HR can negatively regulate PTI in bean plants as we observed overall down-regulation of processes involved in PTI. For instance, the GO enrichment analysis revealed that transcripts classified as transmembrane receptor protein tyrosine kinase activity (GO:0007169) were significantly down-regulated by fungus-inoculation (Fig. S1). Furthermore, transcripts annotated as MAPKKK/MEKK (EC:2.7.11.25) were also under-represented in fungus-inoculated tissue (Table 1). To validate this hypothesis, we searched for bean ESTs with significant similarity to Arabidopsis genes that belonged to those groups (GO:0007169 and EC:2.7.11.25) and known to be involved in PTI. From this selective analysis, we found putative orthologs of the transmembrane receptor *FLS2* (*FLAGELLIN SENSING 2*) and *MKK5* (*MITOGEN-ACTIVATED PROTEIN KINASE KINASE 5*). Owing to their conserved transmembrane LRR (leucine-rich repeat) and STK (serine threonine kinase) domains, several bean ESTs were highly similar to these two Arabidopsis genes (Table S7). We therefore chose the EST with highest similarity to these genes to assess their expression by RT-qPCR. As expected, both *FLS2-like* and *MKK5-like* genes were significantly down-regulated by fungus infection (Fig. 5B).

Although our EST collection was estimated to represent about 25% of the common bean transcriptome [24], it has been valuable in identifying robust responses to the fungal pathogen *C. lindemuthianum* and can be used as reference for comparison among different pathosystems. As more common bean sequences are available (*e.g*., RNA-seq), they can be utilized for a broader analysis of the bean transcriptome allowing for future studies on those common bean genes that have impact on disease resistance and defense against anthracnose.

## Materials and Methods

### EST Library Construction

The construction and annotation of the bean EST database used in this study have been reported previously [24]. Briefly, the bean breeding line SEL 1308 that carries the *Co-4^2^* gene [71] was grown in controlled environment (22°C, 80% relative humidity, and 16h of daily light). and used as source of mRNA to construct EST libraries. Mock-inoculated 10-day old seedling shoots or leaves were used to construct the PVEPSE2 and PVEPLE1 libraries, respectively. Some of the seedlings of the same batch were spray-inoculated with race 73 of *C. lindemuthianum*, which is avirulent on bean plants carrying the *Co4^2^* gene [71] and used to construct the PVEPSE3 library. Inoculum preparation and inoculation methods were conducted as described by Melotto and Kelly [20].Development of HR in the bean/*C. lindemuthianum* pathosystem varies according to the bean cultivar and the fungus race [34], [35]; therefore we collected bean tissue 65 hpi when HR, characterized by minute and limited lesions, was observed on the leaf. As a control, susceptible plants (cultivar Black Magic) were included in the inoculation experiment and these plants showed characteristic anthracnose symptoms starting at five days after inoculation. Inoculation procedure and disease phenotyping were conducted as described elsewhere [72]. ESTs from the PVEPLE1 and PVEPSE2 libraries were analyzed together as they both were constructed with mock-inoculated, control tissue. All sequences reported here are deposited in the NCBI EST database (dbEST) under the accession numbers CB280466 through CB280717, CB539100 through CB543715, CB544073 through CB544239, and CB555925 through CB556132.

### Enzyme Codes and Gene Ontology

Enzyme codes (EC) were attributed to each ESTs based on tBLASTX results against the non-redundant database using the KEGG function of the Blast2GO suite [46], [73]. The two EST collections were analyzed separately. The number of ESTs belonging to each EC was used to assess the relative abundance in each EST collection and calculate statistical significance as described below. Furthermore, each EST sequence was searched against Arabidopsis transcript sequences available at The Arabidopsis Information Resource (TAIR10 database; http://www.arabidopsis.org/) using tBLASTX [74] with a minimum threshold E-value≤1×10^−4^. The AGI (Arabidopsis Genome Initiative) number of the top hit was used as input for assigning Gene Ontology (GO) categories (http://www.geneontology.org/), performing GO enrichment using the Singular Enrichment Analysis (SEA) through AgriGO (http://bioinfo.cau.edu.cn/agriGO/) [49], and assess the representation of GO categories within each EST libraries.

### Gene Expression Analysis and Metabolic Pathways Reconstructions

In order to infer relative expression of genes between mock- and fungus-inoculated tissues, ESTs from each library with significant similarity (E-value≤1×10^−4^) to a single Arabidopsis transcript were clustered, representing the number of observations of a single gene in each library. Library-specific genes that were represented by at least three ESTs, as well as differentially represented genes in each library were identified to infer up- or down-regulation upon fungal infection. Putative Arabidopsis orthologs of significantly regulated bean genes (p≤0.05) were used as input to compare their expression patterns under different stress conditions using Genevestigator (https://www.genevestigator.com/gv/) clustering analysis [75] and to reconstruct metabolic pathways using the Mapman 3.5.1R2 software [76] and KEGG mapping [77].

### Statistical Analysis

Statistical significance of bioinformatic analyses was calculated with the Fisher’s exact test [78]. The Log_10_ of the p-value was used to infer the relative abundance of ECs, GOs, and transcripts identified in EST collections from either mock- or fungus-inoculated tissue [79].

### Reverse Transcriptase – Quantitative PCR (RT-qPCR)

Common bean genotype SEL1308 seedlings growth and fungal inoculations were performed under controlled environmental condition as previously described [24]. Total RNA from control (water-sprayed plants) and inoculated plants was extracted at 65 h post treatment using TRIzol® Reagent Kit (Invitrogen, Carlsbad, CA) according to manufacturer’s instructions. Eight genes were selected (Table S6) for validation of bioinformatic analyses. Two reference genes, *ACT2* and *UNK2*, were used as internal control according to Borges *et al.* [80]. Three biological replicates and three technical replicates were performed for each selected gene.

The amplification reactions were carried out using 100 ng of total RNA, 250 µM of each primer (Table S6), and reagents of the 2X Sensimix™ SYBR & ROX one-step kit (PEQLAB, London, UK). The reaction conditions were set as follows: one cycle for 10 min at 42°C for cDNA synthesis and 10 min at 95°C for reverse transcriptase inactivation and *Taq* DNA polymerase activation, followed by 40 cycles of 15 sec at 95°C, 30 sec at 60°C, and 30 sec at 72°C. A final step of 15 sec at 95°C and 1 min at 60°C was included to obtain the melting curve (0.7°C variation from 60°C to 95°C).

Baseline correction and linear regression analysis of each amplification curve was performed with the LinRegPCR software [81]. The optimal set of data point (Window-of-Linearity) was defined to obtain the threshold and quantification cycle (C_q_) values. The efficiency (E = 10^slope^) was calculated based on the slope line, considering an ideal value range (1.8≤E≤2) and correlation (R≥0.995). The relative expression of target genes and p-values were obtained based on average efficiency and Cq values of target and reference genes using the REST software [82].

## Supporting Information

Figure S1 **Gene Ontology terms in the Biological Processes category uniquely identified in either EST collections using the AgriGO Singular Enrichment Analysis (SEA).** Processes identified only in fungus-inoculated tissue are considered up-regulated by fungal infection (grey bars) and processes identified only in mock-inoculated tissue are considered down-regulated (black bars). Statistical significance was detected with the Fisher’s exact test (p-value≤0.05) and data points are indicated as Log_10_ of the p-value.
(TIF)

Table S1 GO terms using Blast2GO. Gene ontology terms assigned to individual ESTs from two collections using Blast2GO. One collection from two libraries constructed with control, mock-inoculated seedling shoots (PVEPLE1 and PVEPSE2) and another collection constructed with fungus-inoculated seedling shoots (PVEPSE3).
(XLSX)

Table S2 tBLASTX analysis of bean ESTs against the Arabidopsis TAIR10 database. AGI number of each bean EST ortholog was identified and used for grouping orthologs into GO categories using AgriGO Singular Enrichment Analysis.
(XLSX)

Table S3 GO categories identified in each EST collection and number of transcripts assigned to each GO using their putative Arabidopsis orthologs as input for the AgriGO tool.
(XLSX)

Table S4 Relative expression of ESTs and their putative Arabidopsis orthologs. List of bean ESTs and their putative Arabidopsis orthologs. Relative abundance of bean transcripts in each EST collection was determined by counting the number of ESTs with significant similarity (E-value≤1×10^−4^) to the same Arabidopsis transcript model (AGI number). Statistical significance of EST abundance in each library was detected by Fisher’s exact test to infer relative gene expression values (Log_10_ of p-value).
(XLSX)

Table S5 Library-specific ESTs identified three or more times in a single library. The AGI number indicates putative Arabidopsis orthologs of bean transcripts identified with tBLASTX search against the TAIR10 database (E-value≤1×10^−4^ was considered as significant). Statistical significance of the library-specific ortholog abundance was detected with the Fisher’s exact test to obtain p-values. Expression of genes encoding glycosyl hydrolase and DND1 has been validated with qPCR (italic letters).
(DOCX)

Table S6 Bean transcripts and primers for validation of bioinformatics analysis of the EST libraries using RT-qPCR analysis. Putative gene functions were based on the best hit of tBLASTX against the non-redundant database available at NCBI. Primers were designed using the *P. vulgaris* EST sequences. Actin and Unknown genes were used for expression normalization according to procedures described by Borges *et al.* [80].
(DOCX)

Table S7 Bean ESTs with significant similarity to the Arabidopsis genes *FLS2* and *MKK5.*
(DOCX)
