# Supplementary figures and images for: Dissecting Phaseolus vulgaris Innate Immune System against Colletotrichum lindemuthianum Infection
Source: PLoS One. 2012 Aug 17;7(8):e43161. doi: 10.1371/journal.pone.0043161 (PMC3422333; doi:10.1371/journal.pone.0043161)

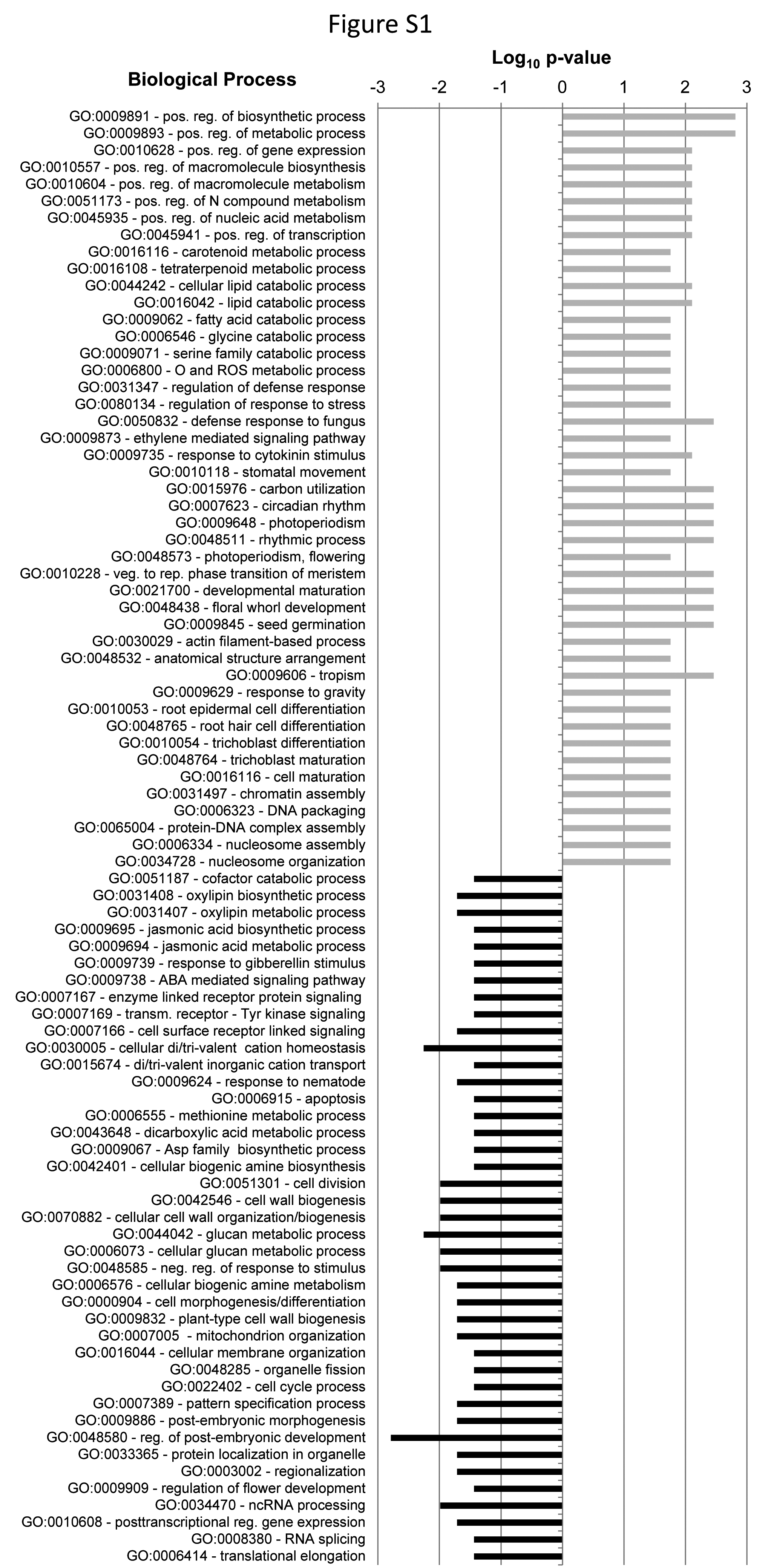

Supplement: Figure S1 — Gene Ontology terms in the Biological Processes category uniquely identified in either EST collections using the AgriGO Singular Enrichment Analysis (SEA). Processes identified only in fungus-inoculated tissue are considered up-regulated by fungal infection (grey bars) and processes identified only in mock-inoculated tissue are considered down-regulated (black bars). Statistical significance was detected with the Fisher’s exact test (p-value≤0.05) and data points are indicated as Log10 of the p-value. (TIF) [file pone.0043161.s001.tif]
