# Supplementary material for: Dissecting Phaseolus vulgaris Innate Immune System against Colletotrichum lindemuthianum Infection
Source: PLoS One. 2012 Aug 17;7(8):e43161. doi: 10.1371/journal.pone.0043161 (PMC3422333; doi:10.1371/journal.pone.0043161)
Supplement: Table S5 — Library-specific ESTs identified three or more times in a single library. The AGI number indicates putative Arabidopsis orthologs of bean transcripts identified with tBLASTX search against the TAIR10 database (E-value≤1×10^−4^ was considered as significant). Statistical significance of the library-specific ortholog abundance was detected with the Fisher’s exact test to obtain p-values. Expression of genes encoding glycosyl hydrolase and DND1 has been validated with qPCR (italic letters). (DOCX) [file pone.0043161.s006.docx]

**Table S5** Library-specific ESTs identified three or more times in a single library. The AGI number indicates putative Arabidopsis orthologs of bean transcripts identified with tBLASTX search against the TAIR10 database (E-value ≤ 1 x 10^-4^ was considered as significant). Statistical significance of the library-specific ortholog abundance was detected with the Fisher’s exact test to obtain p-values. Expression of genes encoding glycosyl hydrolase and DND1 has been validated with qPCR (italic letters).

| **EST identified only in the library from fungus-inoculated tissue (considered as up-regulated transcripts)** | | | | | | | | | |
| --- | --- | --- | --- | --- | --- | --- | --- | --- | --- |
| **AGI number^a^** | | **Annotation** | **Cellular localization** | **Function** | | **E-value** | **Identity** | **p value** | **RE^b^** |
| *AT4G16260* | | *Glycosyl hydrolase, ortholog of the* P. vulgaris *1,3 β-D-glucanase (X53129.1)* | *cell wall, vacuolar membrane* | *defense response to fungus and salt stress; incompatible interaction* | | *1 x 10^-56^* | *66%* | *0.04* | *1.41* |
| AT3G20820 | | Leucine-rich repeat (LRR) | apoplast, cell wall, chloroplast, membrane, plasmodesma | defense response; signal transduction | | 4 x 10^-70^ | 77% | 0.04 | 1.41 |
| AT3G47340 | | glutamine-dependent asparagine synthetase  (ASN1) | cytosol, chloroplast^c^ | defense response to fungus; incompatible interaction; cellular amino acid catabolic process; cellular response to sucrose starvation, absence of light, fructose stimulus, and glucose stimulus | | 2 x 10^-52^ | 72% | 0.04 | 1.41 |
| AT3G16640 | | translationally controlled tumor protein (TCTP) | apoplast, chloroplast, cytoplasm, nucleus, plasma membrane, vacuolar membrane | defense response to bacterium; auxin homeostasis, cell proliferation, embryo development, pollen tube growth, post-embryonic development, regulation of cell growth, response to cadmium ion | | 4 x 10^-30^ | 67% | 0.04 | 1.41 |
| AT2G32060 | | Ribosomal protein L7Ae | cytosolic small ribosomal subunit, nucleus | translation | | 1 x 10^-52^ | 70% | 0.04 | 1.41 |
| AT3G25570 | | Adenosylmethionine decarboxylase | cytosol, apoplast^c^ | spermidine biosynthetic process | | 7 x 10^-59^ | 65% | 0.04 | 1.41 |
| AT4G33580 | | beta carbonic anhydrase 5 | chloroplast | carbon utilization | | 5 x 10^-49^ | 68% | 0.04 | 1.41 |
| AT5G56600 | | actin monomer-binding protein | actin cytoskeleton, cytoplasm, nucleus | actin cytoskeleton organization | | 3 x 10^-50^ | 73% | 0.04 | 1.41 |
| AT2G07706 | | unknown protein | cytosol, chloroplast^c^ | unknown | | 3 x 10^-09^ | 56% | 0.04 | 1.41 |
| ATCG01130 | | hypothetical protein | chloroplast | protein binding | | 4 x 10^-16^ | 42% | 0.04 | 1.41 |
| AT1G74730 | | unknown protein | chloroplast | unknown | | 3 x 10^-38^ | 67% | 0.09 | 1.06 |
| *AT5G15410* | | *'defense, no death' gene (DND1)* | *plasma membrane, mitochondrion^c^* | *plant-type hypersensitive response; nitric oxide mediated signal transduction; potassium and calcium ion transmembrane transport* | | *5 x 10^-69^* | *78%* | *0.09* | *1.06* |
| AT1G06430 | | FtsH protease | chloroplast | ATP catabolic process, PSII associated light-harvesting complex II catabolic process | | 2 x 10^-87^ | 90% | 0.09 | 1.06 |
| AT4G27670 | | small heat shock protein (HSP21) | chloroplast | response to heat, high light intensity, and hydrogen peroxide | | 2 x 10^-23^ | 44% | 0.09 | 1.06 |
| AT5G23120 | | stability and/or assembly factor of photosystem II | chloroplast photosystem II | plastid organization; protein complex assembly; response to cadmium ion | | 1 x 10^-73^ | 90% | 0.09 | 1.06 |
| AT2G46830 | | Circadian clock associated 1 (CCA1) | nucleus | regulation of transcription and protein homodimerization activity circadian rhythm; response to abscisic acid, auxin, cadmium ion, cold, ethylene, gibberellin, jasmonic acid, organic nitrogen, salicylic acid, and salt stress | | 1 x 10^-10^ | 67% | 0.09 | 1.06 |
| AT3G01470 | | homeodomain leucine zipper class I transcription factor | nucleus | leaf morphogenesis; regulation of transcription; response to blue light and salt stress | | 8 x 10^-20^ | 68% | 0.09 | 1.06 |
| AT3G12630 | | E3 ligase activity | nucleus | protein ubiquitination, response to water deprivation | | 3 x 10^-27^ | 87% | 0.09 | 1.06 |
| AT3G14415 | | Aldolase-type TIM barrel family protein | apoplast, chloroplast, nucleus, peroxisome, vacuole | oxidation-reduction process | | 5 x 10^-43^ | 87% | 0.09 | 1.06 |
| AT3G16150 | | N-terminal nucleophile aminohydrolases | cytosol, chloroplast^c^ | asparagine catabolic process via L-aspartate; glycoprotein catabolic process; protein maturation | | 4 x 10^-92^ | 82% | 0.09 | 1.06 |
| AT4G09160 | | SEC14 cytosolic factor family protein | chloroplast | transport | | 4 x 10^-66^ | 73% | 0.09 | 1.06 |
| AT3G18280 | | Bifunctional inhibitor/lipid-transfer protein | endomembrane system | lipid transport | | 8 x 10^-23^ | 56% | 0.09 | 1.06 |
| AT1G07320 | | plastid ribosomal protein L4 | chloroplast, cytosolic ribosome, membrane, nucleus | translation | | 7 x 10^-66^ | 72% | 0.09 | 1.06 |
| AT4G20360 | | RAB GTPase | chloroplast, nucleus | GTP catabolic process; translational elongation | | 3 x 10^-40^ | 93% | 0.09 | 1.06 |
| AT2G41250 | | Haloacid dehalogenase-like hydrolase | mitochondria^c^ | dephosphorylation | | 7 x 10^-59^ | 73% | 0.09 | 1.06 |
| **EST identified only in the library from mock-inoculated tissue (considered as down-regulated transcripts)** | | | | | | | | | |
| **AGI number^a^** | **Annotation** | | **Cellular localization** | **Function** | **E-value** | | **Identity** | **p value** | **RE^b^** |
| AT5G54770 | thiamine biosynthetic gene | | chloroplast, mitochondrion | response to DNA damage stimulus and cold; oxazole, thiazole, and thiamine biosynthetic process | 1 x 10^-89^ | | 90% | 0.02 | -1.70 |
| AT1G17880 | basic transcription factor 3 | | nucleus^c^ | regulation of transcription; response to salt stress | 9 x 10^-54^ | | 85% | 0.13 | -0.87 |
| AT3G26740 | cinnamoyl-CoA reductase (CCR) like | | chloroplast | circadian rhythm | 1 x 10^-26^ | | 47% | 0.13 | -0.87 |
| AT4G37930 | mitochondrial serine hydroxymethyltransferase (SHMT1) | | chloroplast, ribosome, mitochondrion, nucleus, plasma membrane | hypersensitive response; L-serine metabolic process; glycine decarboxylation; photorespiration; response to cadmium ion and cold | 5 x 10^-103^ | | 95% | 0.13 | -0.87 |
| AT4G27440 | light-dependent NADPH:protochlorophyllide oxidoreductase B | | chloroplast | chlorophyll biosynthetic process; oxidation-reduction process; response to ethylene stimulus | 7 x 10^-101^ | | 91% | 0.13 | -0.87 |
| AT5G54190 | light-dependent NADPH:protochlorophyllide oxidoreductase A | | chloroplast | chlorophyll biosynthetic process; oxidation-reduction process; response to ethylene stimulus | 2 x 10^-04^ | | 67% | 0.13 | -0.87 |
| AT1G09640 | elongation factor EF1B | | cytosol, plasma membrane, plasmodesma | translational elongation | 7 x 10^-54^ | | 68% | 0.26 | -0.59 |
| AT3G09200 | Ribosomal protein | | chloroplast, ribosome, nucleolus, nucleus, plasma membrane, plasmodesma | translational elongation; response to cadmium ion, cold, salt stress, and zinc ion | 1 x 10^-82^ | | 88% | 0.26 | -0.59 |
| AT3G27850 | Ribosomal protein | | chloroplast, plastid large ribosomal subunit | translation; defense response to bacterium | 4 x 10^-39^ | | 64% | 0.26 | -0.59 |
| AT5G14320 | Ribosomal protein S13/S18 | | chloroplast, small ribosomal subunit | ribosome biogenesis; translation | 1 x 10^-59^ | | 73% | 0.26 | -0.59 |
| AT4G31700 | putative ribosomal protein S6 | | chloroplast, small ribosomal subunit, nucleolus, plasma membrane, plasmodesma | Growth; rRNA processing; ribosomal small subunit biogenesis; translation | 2 x 10^-37^ | | 94% | 0.26 | -0.59 |
| AT1G16880 | ACT domain-containing protein | | chloroplast | response to cold, sucrose, and light stimulus | 3 x 10^-42^ | | 82% | 0.26 | -0.59 |
| AT1G21065 | unknown protein | | chloroplast | unknown | 3 x 10^-48^ | | 65% | 0.26 | -0.59 |
| AT3G47070 | Thylakoid soluble phosphoprotein | | chloroplast | unknown | 5 x 10^-10^ | | 50% | 0.26 | -0.59 |
| AT3G15690 | Single hybrid motif superfamily | | chloroplast | unknown | 1 x 10^-30^ | | 49% | 0.26 | -0.59 |
| AT4G10300 | RmlC-like cupins | | chloroplast | unknown | 5 x 10^-46^ | | 86% | 0.26 | -0.59 |
| AT5G65470 | O-fucosyltransferase | | endoplasmic reticulum, plasmodesma, mitochondrion, nucleus, peroxisome, plasma membrane^c^ | unknown | 2 x 10^-71^ | | 83% | 0.26 | -0.59 |
| AT3G26060 | periredoxin Q | | chloroplast, plastoglobule | cell redox homeostasis | 1 x 10^-74^ | | 78% | 0.26 | -0.59 |
| AT1G71500 | Rieske (2Fe-2S) domain-containing | | chloroplast | oxidation-reduction process | 8 x 10^-67^ | | 83% | 0.26 | -0.59 |
| AT5G51970 | sorbitol dehydrogenase | | cytosol, plasmodesma | oxidation-reduction process | 4 x 10^-90^ | | 79% | 0.26 | -0.59 |
| AT3G48420 | Haloacid dehalogenase-like hydrolase | | chloroplast | hydrolase activity | 3 x 10^-80^ | | 85% | 0.26 | -0.59 |
| AT4G00430 | plasma membrane intrinsic protein subfamily PIP1 | | plasma membrane | response to water deprivation; water transport | 8 x 10^-71^ | | 84% | 0.26 | -0.59 |
| AT5G61410 | ribulose-5-phosphate-3-epimerase | | chloroplast | carbohydrate metabolic process; embryo development; response to cold and nematode | 4 x 10^-106^ | | 94% | 0.26 | -0.59 |
| AT1G08200 | Putative UPD-D-xylose synthetase | | cytosol, plasma membrane, plasmodesma | nucleotide-sugar biosynthetic process | 8 x 10^-100^ | | 90% | 0.26 | -0.59 |
| AT4G26100 | casein kinase 1 | | plasmodesma | protein phosphorylation | 1 x 10^-98^ | | 95% | 0.26 | -0.59 |
| AT5G60360 | Senescence-associated thiol protease | | vacuole | aging; proteolysis; response to ethylene stimulus | 1 x 10^-75^ | | 67% | 0.26 | -0.59 |
| AT4G35770 | Senescence 1 (SEN1) | | chloroplast | aging; response to jasmonic acid, oxidative stress, and wounding | 5 x 10^-40^ | | 53% | 0.26 | -0.59 |
| AT4G27700 | Rhodanese/Cell cycle control phosphatase | | chloroplast | aging | 2 x 10^-36^ | | 85% | 0.26 | -0.59 |

^a^The bean EST orthologs of each AGI number are listed in Table S2.

^b^RE = relative expression values were obtained by -Log_10_ of p-values for the up-regulated transcripts and by Log_10_ of p-values for the down-regulated genes, according to method described by Zhou *et al*. [79].

^c^Cellular localization was determined using the CelleFPBrowser (<http://bar.utoronto.ca/cell_efp/cgi-bin/cell_efp.cgi>) with minimum or medium confidence.
