# Supplementary material for: Dissecting Phaseolus vulgaris Innate Immune System against Colletotrichum lindemuthianum Infection
Source: PLoS One. 2012 Aug 17;7(8):e43161. doi: 10.1371/journal.pone.0043161 (PMC3422333; doi:10.1371/journal.pone.0043161)
Supplement: Table S6 — Bean transcripts and primers for validation of bioinformatics analysis of the EST libraries using RT-qPCR analysis. Putative gene functions were based on the best hit of tBLASTX against the non-redundant database available at NCBI. Primers were designed using the P. vulgaris EST sequences. Actin and Unknown genes were used for expression normalization according to procedures described by Borges et al. [80]. (DOCX) [file pone.0043161.s007.docx]

**Table S6** Bean transcripts and primers for validation of bioinformatics analysis of the EST libraries using RT-qPCR analysis. Putative gene functions were based on the best hit of tBLASTX against the non-redundant database available at NCBI. Primers were designed using the *P. vulgaris* EST sequences. Actin and Unknown genes were used for expression normalization according to procedures described by Borges *et al.* (2011).

| **EST used for primer design** | **GI number** | **Gene code**  **(accession number)** | **Putative gene function** | **Putative Arabidopsis ortholog** | **Primer sequence** |
| --- | --- | --- | --- | --- | --- |
| PVEPSE2016B09.g | [gi\|59934759\|](http://www.ncbi.nlm.nih.gov/sites/entrez?cmd=Search&db=nucleotide&term=CB540196.1&dopt=GenBank) | PR1-like | Pathogenesis related protein PR1-like | AT2G14580 | F: ACCAATTAGATGGGATGCAACAGT  R: CCCATAAGGACCCCCAGAGT |
| PVEPSE3029H20.g | [gi\|59938056\|](http://www.ncbi.nlm.nih.gov/sites/entrez?cmd=Search&db=nucleotide&term=CB543385.1&dopt=GenBank) | PvPR1 (X61365.1) | MLP-like protein involved in defense response | AT1G24020 | F: AAGGAGATGCTCCACCCAATG  R: CAGGGATTGGCCAGAAGGTA |
| PVEPSE3024G05.g | [gi\|59937438\|](http://www.ncbi.nlm.nih.gov/sites/entrez?cmd=Search&db=nucleotide&term=CB542769.1&dopt=GenBank) | 1,3 β-D-glucanase (X53129.1) | defense response to fungus, incompatible interaction | AT4G16260 | F: AGCAGCTCTGCAAGCACTCA  R: ACGAGCAGTGTCGGCATTG |
| PVEPSE3021D11.g | [gi\|59937251\|](http://www.ncbi.nlm.nih.gov/sites/entrez?cmd=Search&db=nucleotide&term=CB542582.1&dopt=GenBank) | DND1-like | Cyclic nucleotide regulated ion channel | AT5G15410 | F: CATAAAGCGCAGCCAAAGTCT  R: GCGAAGGCACCATGAAAGTAG |
| PVEPSE3029O06.g | [gi\|59938140\|](http://www.ncbi.nlm.nih.gov/sites/entrez?cmd=Search&db=nucleotide&term=CB543469.1&dopt=GenBank) | PAL1-like | Phenylalanine ammonia-lyase; defense response | AT2G37040 | F: ATGGTGCTCCTCTTCCAATTTG  R: TGATTTGCCACAGGCTTACAGA |
| PVEPSE2016G06.g | [gi\|59934802\|](http://www.ncbi.nlm.nih.gov/sites/entrez?cmd=Search&db=nucleotide&term=CB540239.1&dopt=GenBank) | GLP1-like | Germin-like protein; defense response | AT1G72610 | F: GCAGACCTCAAAAGTGCAGATG  R: CAGCTTCAGATAAGCCAGCAAA |
| PVEPSE3010D06.g | [gi59936669\|](http://www.ncbi.nlm.nih.gov/nucest/59936669) | FLS2-like | detection of and defense response to bacterium | AT5G46330 | F:GCCTCACGGTGCTGAACAT  R:TTGTTACGGAGATCCAAGTGCAT |
| PVEPSE3028E14.g | [gi\|59937825](http://www.ncbi.nlm.nih.gov/nucest/59937825?report=est)\| | MKK5-like | mitogen-activated protein kinase (MKK5); defense response; incompatible interaction | AT3G21220 | F:CGAGAGGATCAACACGGACATA  R:CCTGCCCATGTAGAACTCCAA |
| PV_GEa0011B_F08.b1^a^ | [gi\|62703083\|](http://www.ncbi.nlm.nih.gov/sites/entrez?cmd=Search&db=nucleotide&term=CV529679.1&dopt=GenBank) | Actin 1-like | Reference gene | AT3G12110 | F: TGCATACGTTGGTGATGAGG  R: AGCCTTGGGGTTAAGAGGAG |
| NOD_203_C02^a^ | [gi\|62707797\|](http://www.ncbi.nlm.nih.gov/sites/entrez?cmd=Search&db=nucleotide&term=CV534393.1&dopt=GenBank) | Unknown function | Reference gene | AT4g33380 | F: CCAATTCAACCATCCCTCAC  R: AAACTCCTCTGCACCCTCAG |

^a^ ESTs from the collection published by Ramírez *et al.* (2005).
