# Supplementary material for: Dissecting Phaseolus vulgaris Innate Immune System against Colletotrichum lindemuthianum Infection
Source: PLoS One. 2012 Aug 17;7(8):e43161. doi: 10.1371/journal.pone.0043161 (PMC3422333; doi:10.1371/journal.pone.0043161)
Supplement: Table S7 — Bean ESTs with significant similarity to the Arabidopsis genes FLS2 and MKK5. (DOCX) [file pone.0043161.s008.docx]

**Table S7** Bean ESTs with significant similarity to the Arabidopsis genes *FLS2* and *MKK5.*

| ***FLS2*** | | | |
| --- | --- | --- | --- |
| **EST GI#** | **EST clone** | **E-value** | **Identity** |
| CB539697.1 | PVEPSE2009B10.g | 2 x 10^-26^ | 38% |
| CB542106.1 | PVEPSE3010D06.g | 1 x 10^-21^ | 37% |
| CB540685.1 | PVEPSE2022H04.g | 9 x 10^-20^ | 38% |
| CB540686.1 | PVEPSE2022H05.g | 9 x 10^-20^ | 38% |
| CB541284.1 | PVEPSE2031C12.g | 1 x 10^-17^ | 40% |
| CB539540.1 | PVEPSE2006H03.g | 5 x 10^-16^ | 39% |
| CB539780.1 | PVEPSE2010D05.g | 6 x 10^-16^ | 44% |
| CB541695.1 | PVEPSE3001E08.g | 6 x 10^-16^ | 47% |
| CB542240.1 | PVEPSE3012E12.g | 7 x 10^-14^ | 41% |
| CB542751.1 | PVEPSE3024E03.g | 3 x 10^-13^ | 40% |
| CB539467.1 | PVEPSE2005G11.g | 1 x 10^-12^ | 55% |
| CB540952.1 | PVEPSE2026F08.g | 1 X 10^-11^ | 50% |
| CB541410.1 | PVEPSE2033D03.g | 9 x 10^-11^ | 55% |
| CB541794.1 | PVEPSE3003E05.g | 3 x 10^-11^ | 42% |
| CB542885.1 | PVEPSE3026F03.g | 4 x 10^-11^ | 42% |
| CB543266.1 | PVEPSE3028O17.g | 1 x 10^-11^ | 50% |
| CB541230.1 | PVEPSE2030F09.g | 6 x 10^-9^ | 34% |
| CB540851.1 | PVEPSE2025C02.g | 2 x 10^-7^ | 38% |
| CB542597.1 | PVEPSE3021F09.g | 1 x 10^-7^ | 42% |
| CB540132.1 | PVEPSE2015C04.g | 3 x 10^-5^ | 51% |
| CB540902.1 | PVEPSE2025H11.g | 2 x 10^-5^ | 49% |
| CB539426.1 | PVEPSE2005C03.g | 2 x 10^-5^ | 49% |
| CB541657.1 | PVEPSE3017G06.b | 9 x 10^-5^ | 40% |
| ***MKK5*** | | | |
| **EST GI#** | **EST clone** | **E-value** | **Identity** |
| CB543156.1 | PVEPSE3028E14.g | 1 x 10^-83^ | 86% |
| CB541175.1 | PVEPSE2029H08.g | 4 x 10^-16^ | 41% |
| CB541019.1 | PVEPSE2027G04.g | 2 x 10^-13^ | 43% |
| CB542885.1 | PVEPSE3026F03.g | 7 x 10^-13^ | 36% |
| CB541957.1 | PVEPSE3007A06.g | 8 x 10^-11^ | 40% |
| CB541410.1 | PVEPSE2033D03.g | 6 x 10^-9^ | 38% |
| CB543266.1 | PVEPSE3028O17.g | 1 x 10^-7^ | 52% |
| CB543449.1 | PVEPSE3029M12.g | 4 x 10^-7^ | 40% |
| CB540685.1 | PVEPSE2022H04.g | 3 x 10^-6^ | 46% |
| CB540686.1 | PVEPSE2022H05.g | 3 x 10^-6^ | 46% |
